# Inspectors of Lunacy for Scotland

**Published:** 1857-10-01

**Authors:** 


### INSPECTORS OF LUNACY FOR SCOTLAND.

Dr. Browne, of the Crichton Institution, Dumfries, has been selected by the Government as one of the Inspectors of Lunacy under the new Bill. This appointment reflects great credit upon the Government: Dr. Browne may assuredly be pronounced to be the "right man in the right place." Dr. Cox is his colleague.

